# Vitamin B Supplementation and Homocysteine Reduction in Nigerian Children with Nephrotic Syndrome: A Randomized Controlled Trial

**DOI:** 10.12691/jnh-12-1-2

**Published:** 2025-04-24

**Authors:** Bose E. Orimadegun, Adebowale A. Ademola, Adanze O. Asinobi

**Affiliations:** 1 Department of Chemical Pathology, College of Medicine, University of Ibadan, Ibadan, Nigeria; 2 Department of Paediatrics, College of Medicine, University of Ibadan, Ibadan, Nigeria

**Keywords:** nephrotic syndrome, homocysteine, vitamin B6, vitamin B12, folic acid, lipid profile, pediatric nephrology, cardiovascular risk

## Abstract

Nephrotic syndrome (NS) in children is associated with hyperhomocysteinemia, increasing the risk of cardiovascular disease. The deficiency of vitamins B6, B12, and folate contribute to elevated homocysteine levels, yet limited interventional studies have evaluated the effects of vitamin supplementation in pediatric NS patients. This study investigated the effect of folic acid, vitamin B6, and vitamin B12 supplementation on plasma homocysteine levels in Nigerian children with NS. A single-blind, randomized controlled trial was conducted at the University College Hospital, Ibadan, Nigeria. Forty-eight children with NS and plasma homocysteine >10 μmol/L were randomly assigned to receive either daily supplementation (5 mg folic acid, 50 mg vitamin B6, and 1 mg vitamin B12) or placebo for six months. The primary outcome was homocysteine reduction, while secondary outcomes included changes in vitamin levels, renal function, and lipid profiles. At baseline, demographic and biochemical parameters were similar between groups. After six months, the intervention group showed a significant reduction in homocysteine levels (12.8 ± 1.4 μmol/L to 6.9 ± 2.1 μmol/L, p < 0.001), while the control group had minimal change (13.3 ± 1.8 μmol/L to 12.9 ± 1.9 μmol/L, p = 1.000). The intervention group also had greater reductions than the control group in total cholesterol (−13.2 mg/dL vs. −4.9 mg/dL, p < 0.001) and LDL cholesterol (−9.8 mg/dL vs. −3.6 mg/dL, p < 0.001). Renal function parameters improved similarly in both groups. No serious adverse effects were reported, and adherence was 91.7%. Vitamin B supplementation significantly reduced plasma homocysteine and improved lipid profiles in children with NS. These findings suggest potential cardiovascular benefits, warranting further research with larger cohorts and longer follow-up.

## 1. Introduction

Cardiovascular disease remains one of the leading causes of morbidity and mortality worldwide, and even in children, risk factors for future cardiovascular events can manifest early. Among these, elevated plasma homocysteine, a sulphur-containing amino acid, has emerged as a significant modifiable risk factor, strongly linked to endothelial dysfunction, atherosclerosis, and thrombotic complications [1]. Hyperhomocysteinaemia is particularly concerning in children with nephrotic syndrome (NS), a kidney disorder that affects the glomerular filtration barrier, leading to massive proteinuria, hypoalbuminaemia, and oedema. While NS is relatively uncommon, with an annual incidence ranging from 2 to 16.9 per 100,000 children globally [2], its impact on long-term health is profound, particularly in low-resource settings like Nigeria, where focal segmental glomerulosclerosis (FSGS) is frequently observed [3].

Emerging evidence has highlighted a troubling association between NS and elevated homocysteine levels in children. Studies have shown that deficiencies in vitamins B6, B12, and folate, nutrients essential for the proper metabolism of homocysteine, are common in children with NS, possibly due to urinary losses or altered renal metabolism [4]. These deficiencies may exacerbate cardiovascular risk and contribute to the disease’s long-term burden. While observational studies have consistently reported elevated homocysteine levels in NS patients, especially in the paediatric population, interventional research evaluating whether correcting these vitamin deficiencies can lower homocysteine and reduce associated risks remains scarce.

This gap in the literature is concerning, particularly in regions such as sub-Saharan Africa where access to specialist care is limited and cardiovascular outcomes are often poor. Hyperhomocysteinaemia affects up to 57% of Nigerian children with NS [4], yet there are few structured protocols for screening and management. Globally, elevated homocysteine has been associated with nearly a doubling of cardiovascular risk in both adults and children [5], with the economic burden of cardiovascular disease expected to exceed $1 trillion annually by 2030, largely driven by modifiable risk factors [6].

Addressing hyperhomocysteinaemia aligns directly with several Sustainable Development Goals (SDGs), particularly SDG 3, which seeks to ensure healthy lives and promote well-being for all ages. Preventive strategies that reduce cardiovascular risk in children also support national health objectives in Nigeria, where non-communicable diseases are rising in prevalence. Effective, evidence-based interventions, particularly those that are affordable, safe, and scalable, such as micronutrient supplementation, are urgently needed.

This study responds to that call by investigating whether daily oral supplementation with folic acid, vitamin B6, and vitamin B12 can significantly reduce plasma homocysteine levels in Nigerian children with NS. Our central hypothesis is that targeted supplementation will result in meaningful reductions in plasma homocysteine and potentially improve lipid profiles, thereby lowering long-term cardiovascular risk in this vulnerable group.

What distinguishes this study is its focus on a Nigerian paediatric population with confirmed hyperhomocysteinaemia and its use of a rigorous randomised controlled design to evaluate the intervention’s efficacy. Unlike prior research that has focused on adult or dialysis populations [7,8], this trial specifically addresses a paediatric cohort in a low-resource setting, offering a novel contribution to global nephrology and paediatric cardiovascular risk management. The findings have the potential to inform clinical guidelines, influence public health policy, and improve long-term outcomes by integrating nutritional supplementation into the standard care of children with NS.

By addressing a clear gap in the literature and aligning with both clinical priorities and global health goals, this study provides a timely and necessary contribution to improving cardiovascular outcomes in children with chronic kidney disease.

## 2. Methods

### 2.1. Study Design and Setting

This study was designed as a single-blind, randomized controlled trial (RCT) to evaluate the effects of daily oral supplementation with folic acid, vitamin B6, and vitamin B12 on plasma homocysteine levels in Nigerian children diagnosed with NS. The study followed the Consolidated Standards of Reporting Trials (CONSORT) guidelines to ensure methodological rigor and reproducibility [9]. Participants were recruited from the Pediatric Nephrology Clinic and children’s wards of the University College Hospital (UCH) in Ibadan, Nigeria, a major referral center for kidney diseases in southwestern Nigeria. The study was conducted between August 2009 and January 2012.

### 2.2. Study Population

Children younger than 18 years with a confirmed diagnosis of NS based on standard clinical and laboratory criteria were eligible for inclusion in the study. Only those with plasma homocysteine levels greater than 10 μmol/L at baseline were recruited. Participants were required to have no significant renal impairment, defined as a serum creatinine level of more than 3 mg/dL, and no prior use of vitamin supplements. Children with history of hypersensitivity to folate or any other vitamin preparation were excluded. Those with other chronic conditions that could interfere with vitamin metabolism, such as severe malnutrition or liver disease, were also not eligible.

After obtaining informed consent from parents or guardians, all eligible participants underwent a baseline assessment, which included a comprehensive clinical examination and laboratory evaluation. A total of 48 participants were enrolled in the study.

### 2.3. Randomization and Blinding

Participants were randomly assigned to one of two study arms: the vitamin supplementation group or the control group. A computer-generated randomization sequence was used to allocate participants in a 1:1 ratio, ensuring equal distribution between the two groups. Allocation concealment was maintained using sequentially numbered, opaque, sealed envelopes prepared by an independent researcher who was not involved in recruitment or data analysis.

Blinding was applied to laboratory personnel, who remained unaware of the group assignments throughout the study period. However, due to the nature of the intervention, caregivers and clinicians were aware of the treatment allocation. This limited the study to a single-blind design.

### 2.4. Intervention and Control

Children assigned to the intervention group received a daily oral supplementation regimen consisting of 5 mg of folic acid, 50 mg of vitamin B6, and 1 mg of vitamin B12. These supplements were obtained from Vitabiotics Nigeria Ltd. and were provided in identical, pre-packed containers to ensure uniformity. Participants in the control group received a placebo, which was formulated to resemble the vitamin capsules but contained only cellulose fiber.

All participants, regardless of their assigned group, continued to receive standard treatment for NS in accordance with the hospital’s established clinical protocols. This included corticosteroid therapy (prednisolone) and supportive care measures as indicated by the attending pediatric nephrologists.

### 2.5. Follow-Up and Compliance Assessment

Participants were followed up for a total duration of six months, during which they attended scheduled clinic visits every two weeks. At each visit, adherence to the assigned intervention was assessed through caregiver interviews and pill counts. Any participant who missed more than three consecutive doses was classified as non-compliant and excluded from the per-protocol analysis. Clinical evaluations, including measurement of body weight, blood pressure, and assessment of edema, were conducted at each visit. Any complaint, including gastrointestinal symptoms or hypersensitivity reactions, were documented and managed accordingly.

### 2.6. Outcome Measures

The primary outcome measure for this study was the change in plasma homocysteine concentration from baseline to six months following vitamin supplementation. The secondary outcome measures included changes in serum folate, vitamin B6, and vitamin B12 levels, as well as renal function markers such as serum creatinine, urea, and albumin. Additionally, lipid profile parameters, including total cholesterol, low-density lipoprotein cholesterol (LDLc), high-density lipoprotein cholesterol (HDLc), and triglycerides, were analyzed to determine whether vitamin supplementation had any effect on lipid metabolism in children with NS.

### 2.7. Sample Size Determination

The required sample size was calculated based on data from a previous study that examined the effects of vitamin supplementation on homocysteine levels in individuals with renal disorders [10]. A minimum of 32 participants was deemed necessary to detect a mean reduction of 5.0 ± 2.0 μmol/L in homocysteine levels in the intervention group compared to a reduction of 3.0 ± 1.0 μmol/L in the control group. The calculation assumed a power of 90% and a type I error rate of 5%. To account for an anticipated 10% loss to follow-up, a total of 48 participants were enrolled, with 24 participants assigned to each study arm.

### 2.8. Laboratory Analysis

Venous blood samples were collected after an overnight fast at baseline and at the six-month follow-up visit. A total of 5 mL of blood was drawn from each participant and processed immediately. Plasma homocysteine levels were measured using an enzyme immunoassay method as described by Frantzen and colleagues [11]. Serum folate, vitamin B6, and vitamin B12 concentrations were determined using a chemiluminescence immunoassay method. Plasma cholesterol and triglycerides were measured using enzymatic colorimetric methods, while LDLc was calculated using the Friedewald formula [12].

To ensure reliability, all assays were performed in duplicate. Samples were stored at −80°C prior to analysis, and both internal and external quality control measures were applied throughout the study period.

### 2.9. Statistical Analysis

All statistical analyses were conducted using SPSS version 17.0 (SPSS Inc., IL, USA). Descriptive statistics were used to summarize baseline characteristics, with continuous variables presented as means and standard deviations or as medians and interquartile ranges, depending on data distribution. Categorical variables were reported as frequencies and percentages.

Between-group comparisons of baseline characteristics were performed using independent t-tests for normally distributed variables and Mann-Whitney U tests for non-normally distributed data. Paired t-tests and Wilcoxon signed-rank tests were used to evaluate changes in primary and secondary outcome measures within each group.

Multivariate regression analysis was used to identify predictors of homocysteine reduction. The model included age, baseline GFR, serum albumin, and baseline vitamin concentrations as independent variables. The effect sizes were reported as beta coefficients with 95% confidence intervals. A p-value of less than 0.05 was considered statistically significant for all analyses.

### 2.10. Ethical Considerations

This study received ethical approval from the University of Ibadan / University College Hospital (UI/UCH) Ethics Committee (Ref: UI/EC/08/0090). Written informed consent was obtained from parents or guardians before any study procedures were initiated. The research adhered to the ethical principles outlined in the Declaration of Helsinki (2013) regarding the protection of human participants in medical research.

Confidentiality was maintained by assigning unique identification codes to participants, and data were stored securely. Participants and their caregivers were informed that they could withdraw from the study at any time without any consequences.

## 3. Results

### 3.1. Baseline Characteristics of Study Participants

A total of 48 children diagnosed with NS were enrolled and randomized into two groups: the intervention group (n = 24) and the control group (n = 24). The baseline characteristics of the participants were comparable across both groups. The mean age of participants was 104.0 ± 30.8 months (range, 40–140 months). The sex distribution was balanced between the two groups, with the intervention group consisting of 13 males and 11 females, while the control group had 15 males and 9 females. The mean body weight was 27.2 ± 7.8 kg in the intervention group and 26.0 ± 8.7 kg in the control group (p = 0.610). The mean height was 125.7 ± 16.7 cm in the intervention group and 121.5 ± 24.1 cm in the control group (p = 0.482). The mean body mass index (BMI) was 16.9 ± 1.4 kg/m^2^ in the intervention group and 17.3 ± 1.9 kg/m^2^ in the control group (p = 0.450). Overall, the baseline characteristics were largely comparable between the two groups.

### 3.2. Effects of Vitamin Supplementation on Plasma Homocysteine Levels

Plasma homocysteine levels were assessed at baseline and after six months of follow-up in both study groups. At baseline, mean homocysteine levels were 12.8 ± 2.3 μmol/L in the intervention group and 13.1 ± 2.4 μmol/L in the control group (p = 0.632), indicating no significant difference between the groups at the start of the study.

After six months, plasma homocysteine levels significantly decreased in the intervention group to 7.1 ± 1.9 μmol/L, while in the control group, homocysteine levels remained relatively unchanged at 12.7 ± 2.1 μmol/L (p < 0.001). The mean reduction in plasma homocysteine was −5.7 μmol/L (95% CI: −6.5, −4.9, p <0.001) in the intervention group, compared to a negligible reduction of −0.4 μmol/L (95% CI: −0.8, −0.1, p = 0.162) in the control group.

The between-group difference in homocysteine reduction was statistically significant (p <0.001), demonstrating a substantial effect of vitamin supplementation. This reduction in homocysteine levels suggests that the administered combination of folic acid, vitamin B6, and vitamin B12 played a pivotal role in modulating homocysteine metabolism in children with NS. The detailed changes in homocysteine levels over the study period are summarized in Table 2.

**Figure 1.**
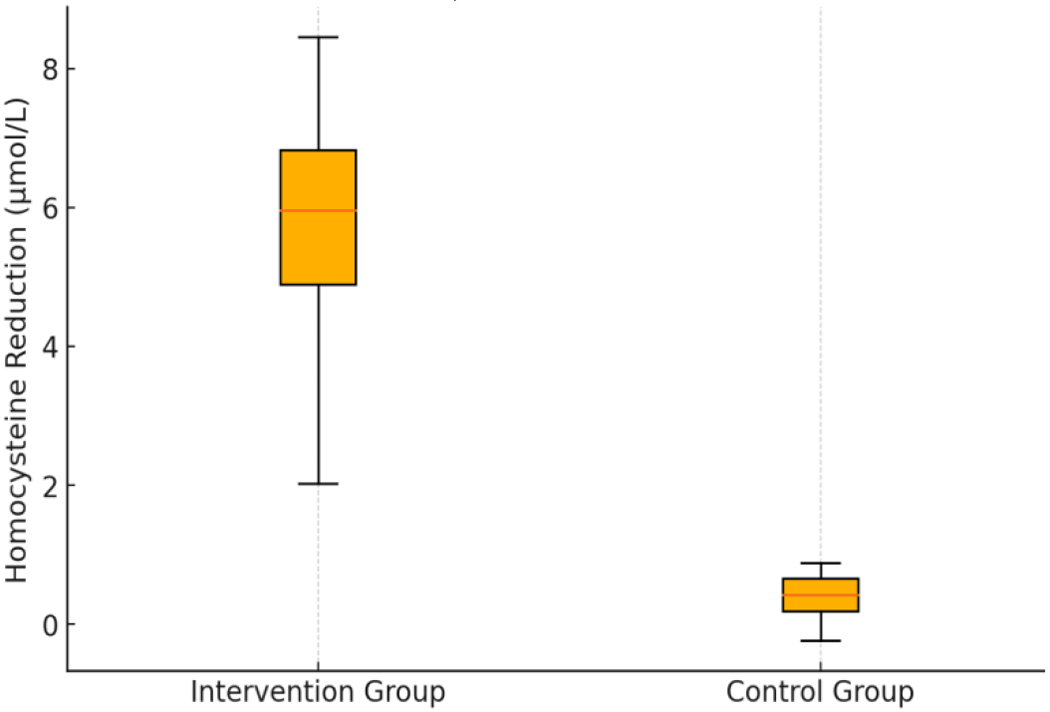
Distribution of Homocysteine Reduction in the Intervention and Control Groups

**Figure 2.**
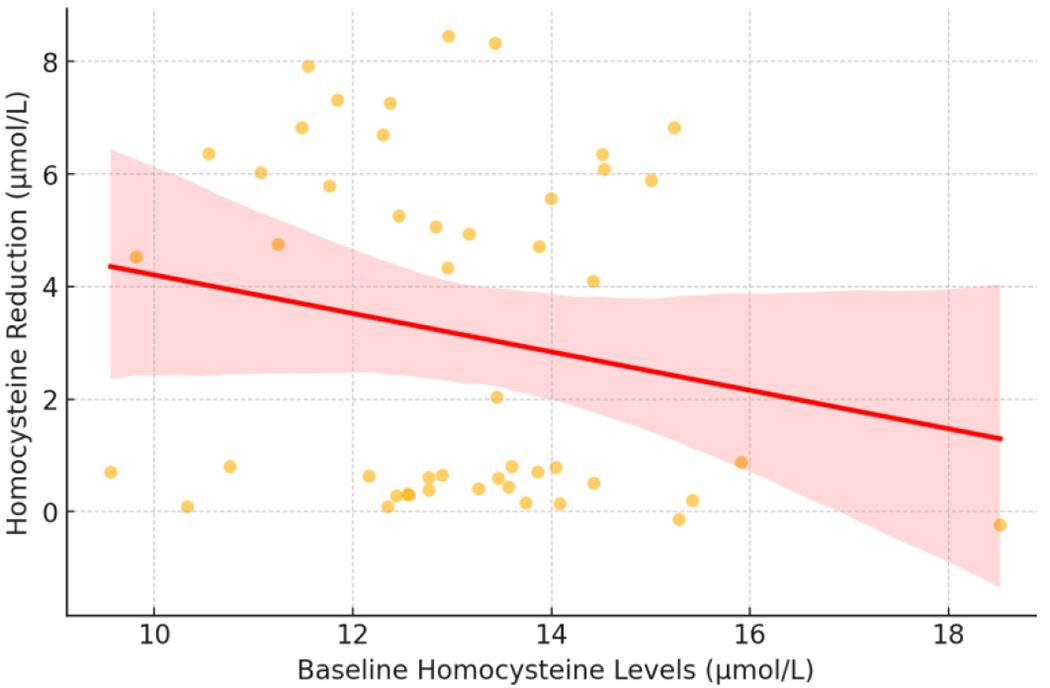
Correlation Between Baseline Homocysteine and Reduction Following Supplementation

Figure 1 presents a box plot that further illustrates homocysteine reduction distribution, while Figure 2 demonstrates the correlation between baseline homocysteine levels and the magnitude of reduction.

### 3.3. Changes in Serum Folate, Vitamin B6, and Vitamin B12 Levels

Serum folate levels increased from 149.0 ± 26.1 ng/mL to 171.3 ± 28.0 ng/mL in the intervention group and from 150.2 ± 17.4 ng/mL to 172.5 ± 18.4 ng/mL in the control group. The mean change was 22.3 ng/mL (95% CI: 20.1, 24.6) and 22.3 ng/mL (95% CI: 20.8, 23.7), respectively (Table 3).

Serum vitamin B6 levels increased from 75.9 ± 7.4 nmol/L to 85.1 ± 8.2 nmol/L in the intervention group and from 69.7 ± 9.6 nmol/L to 81.5 ± 11.5 nmol/L in the control group. Serum vitamin B12 levels rose from 292.6 ± 35.9 pg/mL to 325.9 ± 34.5 pg/mL in the intervention group and from 284.8 ± 46.9 pg/mL to 333.4 ± 46.5 pg/mL in the control group.

### 3.4. Effects of Vitamin Supplementation on Renal Function Parameters

Mean serum creatinine levels declined from 0.71 ± 0.22 mg/dL to 0.53 ± 0.23 mg/dL in the intervention group and from 0.66 ± 0.17 mg/dL to 0.52 ± 0.24 mg/dL in the control group. Serum urea concentrations decreased from 34.6 ± 4.2 mg/dL to 22.0 ± 5.4 mg/dL in the intervention group and from 32.9 ± 5.7 mg/dL to 21.1 ± 6.6 mg/dL in the control group. Serum albumin levels increased from 2.5 ± 0.5 g/dL to 3.8 ± 0.7 g/dL in the intervention group and from 2.8 ± 0.5 g/dL to 4.2 ± 0.7 g/dL in the control group (Table 4).

### 3.5. Effects of Vitamin Supplementation on Lipid Profile

Total cholesterol decreased from 201.3 ± 20.2 mg/dL to 188.1 ± 21.1 mg/dL in the intervention group and from 193.7 ± 23.3 mg/dL to 188.8 ± 23.0 mg/dL in the control group. The mean reduction was −13.2 mg/dL (95% CI: −15.0, −11.5) and −4.9 mg/dL (95% CI: −6.8, −3.0), respectively (Table 5).

LDL cholesterol reduced from 130.1 ± 19.1 mg/dL to 120.3 ± 19.7 mg/dL in the intervention group and from 116.4 ± 23.2 mg/dL to 112.9 ± 23.6 mg/dL in the control group. Conversely, HDL cholesterol levels increased from 50.5 ± 9.4 mg/dL to 55.8 ± 9.8 mg/dL in the intervention group and from 44.7 ± 11.7 mg/dL to 46.7 ± 11.7 mg/dL in the control group.

Triglyceride levels decreased from 135.0 ± 24.2 mg/dL to 116.8 ± 24.7 mg/dL in the intervention group and from 144.4 ± 28.0 mg/dL to 140.2 ± 28.9 mg/dL in the control group. Table 5 summarizes these changes.

### 3.6. Multivariate Regression Analysis of Predictors of Homocysteine Reduction

A multivariate regression model was constructed to identify independent predictors of homocysteine reduction while adjusting for potential confounders, including intervention status, baseline homocysteine levels, renal function markers (serum creatinine, urea, and albumin), and baseline vitamin concentrations (folate, vitamin B6, and vitamin B12).

The analysis revealed that intervention status was a significant predictor of homocysteine reduction. Participants receiving vitamin supplementation showed a β-coefficient of −0.92 (95% CI: −1.75, −0.10, p = 0.029), indicating a strong independent effect of the intervention on lowering homocysteine levels. This finding suggests that the combination of folate, vitamin B6, and vitamin B12 supplementation independently contributed to the reduction in plasma homocysteine levels.

Baseline homocysteine concentration was also a significant predictor, with a β-coefficient of −0.44 (95% CI: −0.80, −0.08, p = 0.041), suggesting that higher initial homocysteine levels were associated with greater reductions following supplementation.

Among renal function parameters, serum creatinine and urea levels did not significantly predict homocysteine reduction. Serum creatinine had a β-coefficient of −0.29 (95% CI: −1.52, 0.94, p = 0.272), while serum urea had a β-coefficient of 0.12 (95% CI: −0.09, 0.33, p = 0.209). Similarly, serum albumin did not show a significant association with homocysteine reduction (β-coefficient: 0.10, 95% CI: −0.05, 0.25, p = 0.175).

Serum folate, vitamin B6, and vitamin-B12 levels exhibited a non-significant trend toward an inverse association with homocysteine reduction. The results of the multivariate regression analysis are summarized in Table 6.

### 3.7. Adverse Effects and Compliance with Vitamin Supplementation

Adherence to supplementation was 91.7% (22 of 24 participants) in the intervention group, with two participants (8.3%) non-adherent due to difficulty swallowing the capsules (n = 1) and parental concerns (n = 1). No serious adverse effects were reported. Mild gastrointestinal discomfort was observed in three participants (12.5%), presenting as transient nausea (n = 2) and mild diarrhea (n = 1). Symptoms resolved without intervention. No adverse effects occurred in the control group. All intervention group participants completed the study.

## 4. Discussion

### 4.1. Discussion of Findings

The findings of this study provide compelling evidence on the efficacy of vitamin supplementation in managing hyperhomocysteinemia among children with NS. The significant reduction in plasma homocysteine levels observed in the intervention group underscores the potential therapeutic role of vitamin B supplementation in this population.

Hyperhomocysteinemia has been identified as a prevalent concern in pediatric NS patients. A study conducted in Nigeria reported that 57.1% of children with NS exhibited elevated homocysteine levels, with mean concentrations significantly higher than those of healthy controls [4]. This aligns with our baseline findings, where mean homocysteine levels were elevated in both the intervention and control groups.

Beyond homocysteine reduction, our study observed favorable changes in lipid profiles among participants receiving vitamin supplementation. Total cholesterol, LDL cholesterol, and triglyceride levels decreased, while HDL cholesterol levels increased in the intervention group. These findings are noteworthy, as dyslipidemia is a common complication in NS, contributing to an increased risk of cardiovascular disease. The observed lipid-modifying effects of vitamin supplementation may be attributed to the role of B vitamins in lipid metabolism and their potential to improve endothelial function [1,13].

Renal function parameters also improved over the six-month period, with reductions in serum creatinine and urea levels, alongside increases in serum albumin concentrations. While both groups exhibited these improvements, the changes were more pronounced in the intervention group. This suggests that factors beyond vitamin supplementation, such as standard medical management and dietary interventions, may have contributed to the observed renal function enhancements.

### 4.2. Comparison of Study Findings with Existing Literature

The findings of this study align with existing literature regarding the prevalence of hyperhomocysteinemia in children with NS. Previous research has demonstrated that hyperhomocysteinemia is common among pediatric NS patients, with studies indicating elevated homocysteine levels in this population [5]. This consistency underscores the significance of addressing hyperhomocysteinemia in the management of NS. The marked reduction in homocysteine concentrations following vitamin supplementation in our study is consistent with existing literature, which demonstrates that supplementation with folic acid, vitamin B6, and vitamin B12 effectively lowers homocysteine levels [1]. Vitamin B supplementation significantly reduces plasma homocysteine levels, which is linked to better endothelial health, though direct improvements in vascular function remain inconclusive.

In contrast, some studies have reported that while vitamin supplementation effectively reduces homocysteine levels, it does not necessarily translate to improved clinical outcomes, such as reduced cardiovascular events [5]. For instance, the FAVORIT trial demonstrated that high-dose folic acid, vitamin B6, and B12 supplementation decreased homocysteine levels but did not improve cardiovascular outcomes in patients with chronic kidney disease [8]. This discrepancy may be due to differences in study populations, baseline homocysteine levels, and the presence of other risk factors [7].

### 4.3. Implications of Findings

The implications of these findings are multifaceted. From a clinical perspective, incorporating vitamin B supplementation into the management plan for pediatric NS patients could serve as a viable strategy to mitigate hyperhomocysteinemia and its associated cardiovascular risks. Given the well-established association between elevated homocysteine levels and endothelial dysfunction, reducing homocysteine concentrations may confer vascular protective effects, thereby improving long-term outcomes for these patients [14].

Furthermore, the lipid-lowering effects observed with vitamin supplementation may offer an adjunctive benefit in managing dyslipidemia in NS patients. This is particularly relevant considering that traditional lipid-lowering therapies, such as statins, may have limitations or potential adverse effects in the pediatric population [4]. Therefore, vitamin B supplementation could represent a safer alternative or complementary approach to lipid management in this cohort.

From a public health and policy standpoint, these findings highlight the importance of nutritional interventions in the comprehensive care of children with NS. Implementing guidelines that recommend routine assessment of homocysteine and vitamin B levels, followed by appropriate supplementation, could enhance the standard of care and potentially reduce the burden of cardiovascular complications in this vulnerable population.

### 4.4. Strengths and Limitations of Findings

This study has several strengths that enhance its validity and clinical relevance. As a randomized controlled trial (RCT), it provides a rigorous methodological framework for evaluating the effect of vitamin supplementation on plasma homocysteine levels in children with NS. The use of computer-generated randomization with allocation concealment minimized selection bias, while blinded laboratory personnel reduced the risk of measurement bias, ensuring reliable biochemical assessments. The study also employed well-defined eligibility criteria, selecting a homogenous population with elevated plasma homocysteine levels and no severe renal impairment, thereby improving internal validity. Additionally, the six-month follow-up period with periodic assessments strengthened the study’s ability to capture meaningful changes over time, and the high adherence rate (91.7%) in the intervention group underscores the feasibility of vitamin supplementation in this population.

Another key strength lies in the comprehensive biochemical evaluation of homocysteine, vitamin status, renal function, and lipid profiles, providing a holistic understanding of the intervention’s effects. The use of validated laboratory techniques, including enzyme immunoassay and chemiluminescence immunoassay, ensured high analytical precision. The application of multivariate regression analysis further enhanced the robustness of the findings by adjusting for potential confounders. Importantly, this study addresses a critical gap in interventional research on hyperhomocysteinemia in pediatric NS, particularly in resource-limited settings like Nigeria. These findings contribute valuable insights that may inform clinical guidelines and public health strategies, emphasizing the role of nutritional interventions in managing cardiovascular risk factors in children with NS.

However, it is essential to acknowledge the limitations of this study. The sample size was relatively small, which may limit the generalizability of the findings. The study duration was six months; longer follow-up periods are necessary to assess the sustained effects of vitamin supplementation on cardiovascular and renal outcomes. Additionally, the study did not assess dietary intake, which could influence homocysteine levels and nutrient status. Finally, while the study observed improvements in surrogate markers such as homocysteine levels and lipid profiles, it did not directly measure clinical outcomes like cardiovascular events.

Future research should focus on larger, multicenter trials with extended follow-up periods to confirm these findings and evaluate the long-term benefits of vitamin B supplementation in children with NS. Studies should also aim to elucidate the mechanisms underlying the observed improvements in renal function parameters and explore the potential renoprotective effects of such interventions. Additionally, assessing dietary intake and controlling nutritional status would provide a more comprehensive understanding of the factors influencing homocysteine levels and the efficacy of supplementation.

### 4.5. Conclusion

Nephrotic syndrome in children is associated with an increased risk of cardiovascular disease, partly due to dyslipidemia and elevated homocysteine levels. Addressing modifiable risk factors through interventions like vitamin B supplementation could significantly impact the long-term health outcomes of these patients. This study contributes to the growing body of evidence supporting the role of vitamin B supplementation in managing hyperhomocysteinaemia and improving cardiovascular risk profiles in pediatric NS.

## Figures and Tables

**Table 1.** Baseline Characteristics of Study Participants

	Group		
Characteristic	Intervention (Mean ± SD)	Control (Mean ± SD)	P
Age (months)	104.0 ± 30.8	104.0 ± 30.8	0.086
Sex (M/F)	13/11	15/9	-
Weight (kg)	27.2 ± 7.8	26.0 ± 8.7	0.610
Height (cm)	125.7 ± 16.7	121.5 ± 24.1	0.482
BMI (kg/m^2^)	16.9 ± 1.4	17.3 ± 1.9	0.450

**Table 2.** Changes in Plasma Homocysteine Levels from Baseline to Six Months

Parameter	Intervention Group	Control Group	p-value
Baseline	12.8 ± 2.3	13.1 ± 2.4	0.632
6 Months	7.1 ± 1.9	12.7 ± 2.1	<0.001
Mean Change (95% CI)	−5.7 (−6.5, −4.9)	−0.4 (−0.8, −0.1)	<0.001

Values are in μmol/L

**Table 3.** Effects of Vitamin Supplementation on Serum Folate, Vitamin B6, and Vitamin B12 Levels

Parameter	Intervention Group	Control Group
Baseline Folate (ng/mL)	149.0 ± 26.1	150.2 ± 17.4
6-Month Folate (ng/mL)	171.3 ± 28.0	172.5 ± 18.4
Mean Change (ng/mL)	22.3	22.3
95% CI of Change	[20.1, 24.6]	[20.8, 23.7]
P-Value	0.967	-
Baseline Vitamin B6 (nmol/L)	75.9 ± 7.4	69.7 ± 9.6
6-Month Vitamin B6 (nmol/L)	85.1 ± 8.2	81.5 ± 11.5
Mean Change (nmol/L)	9.1	11.8
95% CI of Change	[7.2, 11.1]	[9.8, 13.8]
P-Value	0.059	-
Baseline Vitamin B12 (pg/mL)	292.6 ± 35.9	284.8 ± 46.9
6-Month Vitamin B12 (pg/mL)	325.9 ± 34.5	333.4 ± 46.5
Mean Change (pg/mL)	33.3	48.7
95% CI of Change	[27.8, 38.8]	[43.4, 54.0]
P-Value	<0.001	-

**Table 4.** Effects of Vitamin Supplementation on Renal Function Parameters

Parameter	Intervention Group	Control Group
Baseline Serum Creatinine (mg/dL)	0.71 ± 0.22	0.66 ± 0.17
6-Month Serum Creatinine (mg/dL)	0.53 ± 0.23	0.52 ± 0.24
Mean Change (mg/dL)	−0.18	−0.14
95% CI of Change	[−0.22, −0.14]	[−0.20, −0.08]
P-Value	0.271	-
Baseline Serum Urea (mg/dL)	34.6 ± 4.2	32.9 ± 5.7
6-Month Serum Urea (mg/dL)	22.0 ± 5.4	21.1 ± 6.6
Mean Change (mg/dL)	−12.6	−11.8
95% CI of Change	[−14.9, −8.4]	[−13.2, −10.4]
P-Value	0.018	-
Baseline Serum Albumin (g/dL)	2.5 ± 0.5	2.8 ± 0.5
6-Month Serum Albumin (g/dL)	3.8 ± 0.7	4.2 ± 0.7
Mean Change (g/dL)	1.2	1.4
95% CI of Change	[1.0, 1.5]	[1.1, 1.6]
P-Value	0.415	-

**Table 5.** Changes in Lipid Profile Following Vitamin Supplementation

Parameter	Intervention Group	Control Group
Baseline Total Cholesterol (mg/dL)	201.3 ± 20.2	193.7 ± 23.3
6-Month Total Cholesterol (mg/dL)	188.1 ± 21.1	188.8 ± 23.0
Mean Change (mg/dL)	−13.2	−4.9
95% CI of Change	[−15.0, −11.5]	[−6.8, −3.0]
P-Value	<0.001	-
Baseline LDL Cholesterol (mg/dL)	130.1 ± 19.1	116.4 ± 23.2
6-Month LDL Cholesterol (mg/dL)	120.3 ± 19.7	112.9 ± 23.6
Mean Change (mg/dL)	−9.8	−3.6
95% CI of Change	[−12.0, −7.7]	[−5.3, −1.8]
P-Value	<0.001	-
Baseline HDL Cholesterol (mg/dL)	50.5 ± 9.4	44.7 ± 11.7
6-Month HDL Cholesterol (mg/dL)	55.8 ± 9.8	46.7 ± 11.7
Mean Change (mg/dL)	5.3	2
95% CI of Change	[4.5, 6.2]	[1.2, 2.8]
P-Value	<0.001	-
Baseline Triglycerides (mg/dL)	135.0 ± 24.2	144.4 ± 28.0
6-Month Triglycerides (mg/dL)	116.8 ± 24.7	140.2 ± 28.9
Mean Change (mg/dL)	−18.2	−4.2
95% CI of Change	[−21.4, −15.1]	[−8.4, −0.1]
P-Value	<0.001	-

**Table 6.** Multivariate Regression Analysis for Predictors of Homocysteine Reduction

Predictor Variable	β	95% CI	p-value
Intervention Status	−0.92	(−1.75, −0.10)	0.029
Baseline Homocysteine (μmol/L)	−0.44	(−0.80, −0.08)	0.041
Serum Creatinine (mg/dL)	−0.29	(−1.52, 0.94)	0.272
Serum Urea (mg/dL)	0.12	(−0.09, 0.33)	0.209
Serum Albumin (g/dL)	0.10	(−0.05, 0.25)	0.175
Serum Folate (ng/mL)	−0.07	(−0.18, 0.04)	0.108
Serum Vitamin B6 (nmol/L)	−0.06	(−0.14, 0.02)	0.091
Serum Vitamin B12 (pg/mL)	−0.04	(−0.11, 0.03)	0.084
